# A Step Backward and Forward in an Age-Friendly University Initiative: Adapting a Campus Learning Partnership

**DOI:** 10.1093/geroni/igab046.1498

**Published:** 2021-12-17

**Authors:** Ramraj Gautam, Karen Devereaux Melillo, Carol McDonough

**Affiliations:** UMass Lowell, Lowell, Massachusetts, United States

## Abstract

The 5-campus UMass system received designation as an Age-Friendly University (AFU) in 2019. AFU Principle 1 highlights the importance of involving older adults in University activities. UMass Lowell’s Center for Gerontology Research and Partnerships collaborated with the Learning in Retirement Association (LIRA) in Spring 2020 to offer aging-related courses around healthy aging. However, due to COVID-19, these were canceled and are re-scheduled for Spring 2021 via Zoom. The paper will describe the process of selecting course offerings with LIRA and the subsequent cancellation/rescheduling process and adaptation needed. A course will focus on AFU initiative and the opportunities and challenges at UMass Lowell. Likewise, the other course will offer a session on technology and aging where age-based digital divide and strategies for reducing it will be discussed. This paper will reflect on how the collaboration with LIRA and course selection process relates to the AFU principles 1, 5 and 9.

